# Tetramethylpyrazine Protects Against Chronic Hypobaric Hypoxia-Induced Cardiac Dysfunction by Inhibiting CaMKII Activation in a Mouse Model Study

**DOI:** 10.3390/ijms26010054

**Published:** 2024-12-24

**Authors:** Pengfei Zhang, Huifang Deng, Xiong Lan, Pan Shen, Zhijie Bai, Chaoji Huangfu, Ningning Wang, Chengrong Xiao, Yehui Gao, Yue Sun, Jiamiao Li, Jie Guo, Wei Zhou, Yue Gao

**Affiliations:** 1 Beijing Institute of Radiation Medicine, Beijing 100850, China; sdyxyzpf@163.com (P.Z.); denghuifang1998@163.com (H.D.); 19173264403@163.com (X.L.); spluto@foxmail.com (P.S.); 13126616096@163.com (Z.B.); hfcj0370@foxmail.com (C.H.); wnnbeijing@163.com (N.W.); 13021289878@163.com (C.X.); g1076192612@163.com (Y.G.); 13897285632@163.com (Y.S.); lijm83@mail2.sysu.edu.cn (J.L.); 19922609442@163.com (J.G.); 2 State Key Laboratory of Kidney Diseases, Chinese PLA General Hospital, Beijing 100853, China

**Keywords:** hypobaric hypoxia, tetramethylpyrazine, cardiac dysfunction, CaMKII

## Abstract

Chronic exposure to high altitudes causes pathophysiological cardiac changes that are characterized by cardiac dysfunction, cardiac hypertrophy, and decreased energy reserves. However, finding specific pharmacological interventions for these pathophysiological changes is challenging. In this study, we identified tetramethylpyrazine (TMP) as a promising drug candidate for cardiac dysfunction caused by simulated high-altitude exposure. By utilizing hypobaric chambers to simulate high-altitude environments, we found that TMP improved cardiac function, alleviated cardiac hypertrophy, and reduced myocardial injury in hypobaric hypoxic mice. RNA sequencing showed that TMP also upregulated heart-contraction-related genes that were suppressed by hypobaric hypoxia exposure. Mechanistically, TMP inhibited hypobaric hypoxia-induced cardiac Ca^2+^/calmodulin-dependent kinase II (CaMKII) activation and exerted cardioprotective effects by inhibiting CaMKII. Our data suggest that TMP application may be a promising approach for treating high-altitude-induced cardiac dysfunction, and they highlight the crucial role of CaMKII in hypobaric hypoxia-induced cardiac pathophysiology.

## 1. Introduction

Over 140 million individuals around the world reside at altitudes above 2500 m, where chronic hypobaric hypoxia poses challenges to the health of highland residents. Chronic exposure to high altitude causes cardiac pathological changes, as indicated by investigative studies. The cardiac stroke volume declined to 80% of its value at sea level when measured at a high altitude [1]. The stroke volume reached its maximum capacity earlier under hypoxia compared to normoxia [2]. The decreased stroke volume may be owing to the reduced left ventricular (LV) end-diastolic volume. However, the left ventricular ejection fraction remained largely unchanged upon ascending to the plateau [1]. Thus, the left ventricular diastolic function is impaired, while the systolic function is largely retained under high-altitude hypoxia [3,4]. The pathological changes in the right heart are more prominent than those in the left heart. These changes manifest as a dilated right ventricular (RV) cavity, hypertrophy, and reduced right ventricular function [5,6,7]. Hypoxic pulmonary hypertension, caused by the contraction of pulmonary arterioles, may partially account for the pathological changes in the right heart [8]. Thus, right ventricular hypertrophy is a consequence rather than a direct effect of hypoxia [9]. In summary, chronic high-altitude exposure results in cardiac dysfunction, RV dilation, RV hypertrophy, compromised energy reserves, and pulmonary hypertension [4,7,10]. However, there are still no effective treatment strategies for cardiac dysfunction induced by high-altitude exposure.

Tetramethylpyrazine/ligustrazine (TMP) is among the characteristic alkaloids isolated from Ligusticum chuanxiong Hort., a medicinal herb with a long history of common use in China. TMP exerts therapeutic effects against a variety of diseases, such as myocardial ischemia, atherosclerosis, ischemic cerebrovascular diseases, Alzheimer’s disease, spinal cord injury, and hepatic fibrosis [11,12,13,14,15]. Moreover, TMP has been demonstrated to alleviate hypoxia-induced injuries: TMP ameliorates hypoxia-induced cardiomyocyte apoptosis through inhibiting the HIF-1α/JNK/p38 signaling mediated increase in pro-apoptotic proteins [16], provides synergistic protection with borneol for brain microvascular endothelium cells injured by hypoxia [17], and exhibits neuroprotective effects on primary hippocampal neurons exposed to hypoxia [12]. However, whether TMP ameliorates the cardiac dysfunction induced by hypobaric hypoxia exposure remains largely elusive.

CaMKII (Ca^2+^/calmodulin-dependent kinase II), a serine/threonine protein kinase, promotes the phosphorylation of its substrates upon activation. CaMKII is activated and phosphorylated at threonine 287 by the binding of calcified calmodulin (Ca^2+^/CaM) to its regulatory domain [18]. Thus, the increased intracellular Ca^2+^ is the main reason for CaMKII activation. The isoform δ is the predominant isoform of CaMKII in the heart [19]. The excessive activation of CaMKII is associated with various cardiovascular diseases, such as heart failure [20], myocardial hypertrophy [21], myocardial ischemic diseases [22,23,24,25], and arrhythmia [26,27]. Inhibition or ablation of CaMKII significantly alleviates these diseases predominantly by improving cardiac function or alleviating cardiac hypertrophy. For example, inhibiting CaMKII restores Ca^2+^ load in the sarcoplasmic reticulum and, thus, improves myocardial contractility in failing human myocardium [28,29]. Ablation of CaMKII in mice significantly relieves stress overload-induced myocardial hypertrophy by preventing sarcoplasmic reticulum Ca^2+^ leakage or by dephosphorylating histone deacetylase 4 (HDAC4) [20,30]. Additionally, CaMKII is also involved in other intracellular processes such as inflammation, myocyte apoptosis, and metabolism [31,32,33]. Recent studies demonstrated that CaMKII is activated under hypoxia to increase sodium and potassium current, which may contribute to hypoxic ventricular arrhythmia [34,35]. Thus, CaMKII is activated by hypoxia and contributes to a pathological cardiac state.

This study aimed to investigate the therapeutic effects of TMP on cardiac dysfunction induced by chronic hypobaric hypoxia in simulated high-altitude environments and to explore the potential role of CaMKII in this process. Our study supports the notion that TMP is a promising candidate for treating high-altitude-induced cardiac dysfunction.

## 2. Results

### 2.1. Chronic Hypobaric Hypoxia Impairs Right Ventricular Function

**Figure 1:**
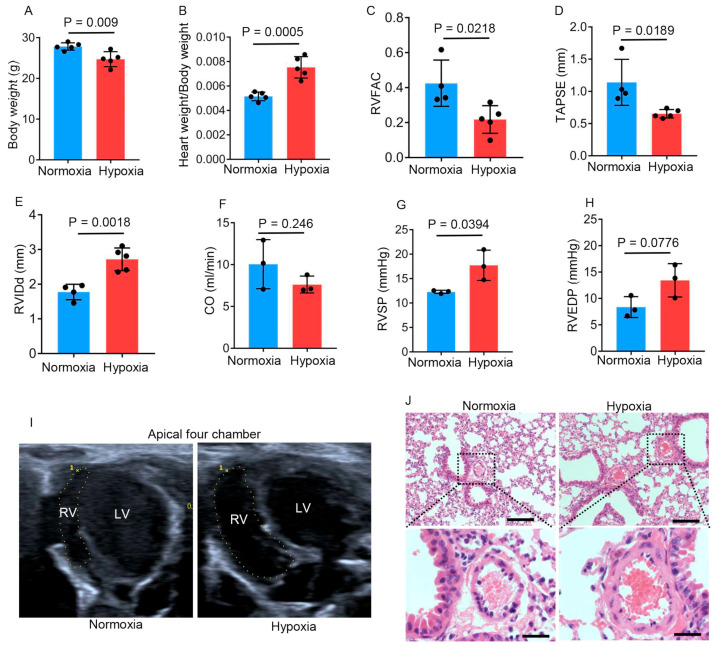
Chronic hypobaric hypoxia impairs right ventricular function. Six-week-old male mice were kept in a hypobaric chamber with pressure at about 47 kPa (hypoxia) or in a sea-level environment (normoxia) for four weeks. The following data were collected: (**A**) Body weights of normoxic and hypobaric hypoxic mice (*n* = 5 per group); (**B**) Heart weight/body weight ratios of normoxic and hypobaric hypoxic mice (*n* = 5 per group); (**C**–**E**) Measurements of the RVFAC (**C**), TAPSE (**D**), and RVIDd (**E**) by means of echocardiography in normoxic (*n* = 4) and hypobaric hypoxic (*n* = 5) mice; (**F**) Measurements of CO by means of echocardiography in normoxic and hypobaric hypoxic mice (*n* = 3 per group); (**G**,**H**) The RVSP (**G**) and RVEDP (**H**) measured by means of right heart catheterization in normoxic and hypobaric hypoxic mice (*n* = 3 per group); (**I**) Representative images of end-diastolic areas of the right ventricle on an apical four-chamber view; (**J**) Representative images of pulmonary arterioles in normoxic and hypobaric hypoxic mice. The vascular wall thickness was slightly increased in hypobaric hypoxic mice. Scale bars in upper images: 200 μm; scale bars in lower images: 50 μm. Data are shown as the mean ± s.d. Statistical analyses in (**A**–**H**) were performed with unpaired Student’s *t*-tests.

To study the effect of high-altitude exposure on cardiac function, we established a chronic hypobaric hypoxic mouse model by housing adult male (6-week-old) C57BL/6 mice in a hypobaric chamber (47 kPa, equivalent to the atmospheric pressure at 6000 m) for 4 weeks. Normoxic control adult mice were maintained in a sea-level environment for a similar period. Body weights, echocardiographic parameters, and hemodynamics were collected following the 4-week exposure. Compared to the normoxic mice (27.84 ± 0.40 g), the hypobaric hypoxic mice had a reduced body weight (24.72 ± 0.82 g), as determined by an unpaired Student’s *t*-test (*p* = 0.009) (Figure 1A). We further measured the heart weight/body weight ratio (HW/BW), an indicator of the relative size of the heart. The normoxic mice had an average HW/BW of 0.0052 ± 0.00016. Meanwhile, the hypobaric hypoxic mice had an increased average HW/BW (0.0075 ± 0.00040). The difference in HW/BW between the two groups was significant (*p* = 0.0005; unpaired Student’s *t*-test) (Figure 1B). To evaluate the influence of hypobaric hypoxia on heart function, we measured the right ventricular fractional area change (RVFAC), a surrogate measurement of the RV ejection fraction, on an apical four-chamber view via echocardiography. Compared to the normoxic mice (0.43 ± 0.066), the hypobaric hypoxic mice had a decreased RVFAC (0.22 ± 0.035), as determined by an unpaired Student’s *t*-test (*p* = 0.0218), indicating that chronic hypobaric hypoxia impaired right ventricular systolic function (Figure 1C,I). We further detected the tricuspid annular plane systolic excursion (TAPSE) on an apical four-chamber view, another indicator of RV systolic function. The average TAPSE of the normoxic mice was 1.14 ± 0.18 mm. Compared to the normoxic mice, the hypobaric hypoxic mice had a significantly decreased average TAPSE of 0.65 ± 0.029 mm (*p* = 0.0189, unpaired Student’s *t*-test) (Figure 1D). We further detected the end-diastolic right ventricular internal diameter (RVIDd), which is an indicator of the degree of the right ventricular enlargement, on the parasternal short-axis section view. The average RVIDd of the hypobaric hypoxic mice was 2.72 ± 0.15 mm, which was longer than that of the normoxic mice (1.78 ± 0.11 mm), as analyzed by unpaired Student’s *t*-test (*p* = 0.0018) (Figure 1E). Thus, hypobaric hypoxic exposure resulted in the enlarged right ventricle. The cardiac output (CO) showed no difference between the normoxic mice and the hypobaric hypoxic mice, as determined by unpaired Student’s *t*-test (*p* = 0.246) (Figure 1F). We further measured the right ventricular systolic pressure (RVSP) and right ventricular end-diastolic pressure (RVEDP) of the normoxic mice and the hypobaric hypoxic mice by means of right heart catheterization. The hypobaric hypoxic mice exhibited elevated RVSP (17.72 ± 1.79 mmHg) compared to the normoxic mice (12.28 ± 0.19 mmHg), as analyzed by unpaired Student’s *t*-test (*p* = 0.0394) (Figure 1G). The elevated RVSP reflects the increased right ventricular afterload of the hypobaric hypoxic group. However, the RVEDP showed no significant difference between the normoxic mice and the hypobaric hypoxic mice (*p* = 0.0776, unpaired Student’s *t*-test) (Figure 1H). Hypobaric hypoxic mice also exhibited pulmonary vascular remodeling compared with the normoxic control after 4 weeks of hypobaric hypoxic exposure (Figure 1J). Taken together, these findings indicate that hypobaric hypoxia impairs right ventricular function and results in pulmonary vascular remodeling.

### 2.2. TMP Improves Cardiac Function and Alleviates Myocardial Injury in Hypobaric Hypoxic Mice

**Figure 2:**
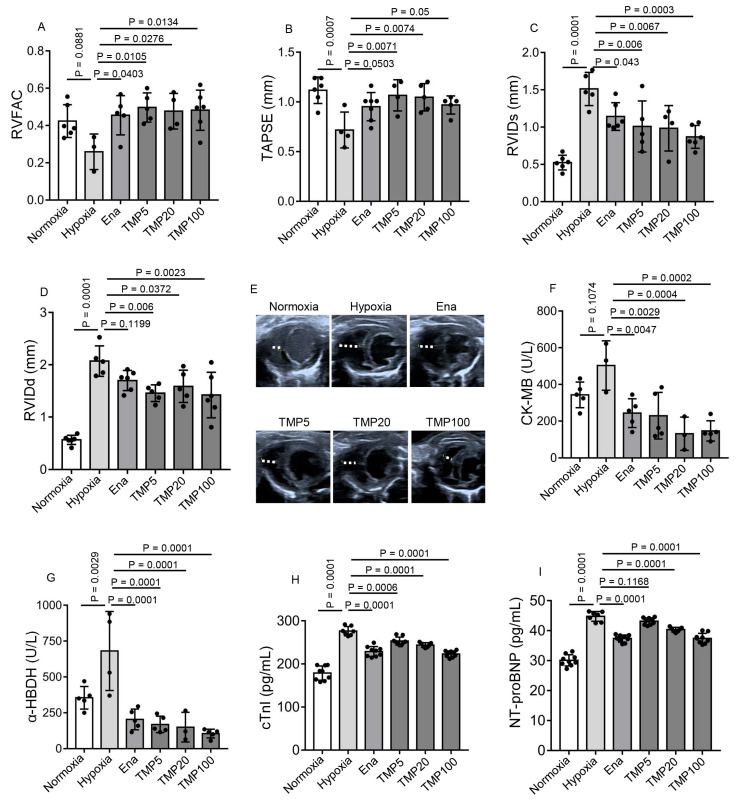
TMP improves cardiac function and alleviates myocardial injury in hypobaric hypoxic mice. Mice were treated with saline, enalapril (20 mg/kg), or TMP at different doses (5, 20, or 100 mg/kg) by means of daily gavage for 4 weeks during hypobaric hypoxia exposure, named a Hypoxia group, Ena group, TMP5 group, TMP20 group, and TMP100 group, respectively; Normoxic mice treated with saline for 4 weeks served as a control, named a Normoxia group. The following data were collected after the 4-week experiment: (**A**–**D**) Measurements of the RVFAC (**A**), TAPSE (**B**), RVIDs (**C**), and RVIDd (**D**) by means of echocardiography in these groups (*n* = 3 to 6 per group); (**E**) Representative images of RVIDd measurement on a parasternal short-axis section view in normoxic and hypobaric hypoxic mice in these groups; (**F**,**G**) Measurements of serum CK-MB (**F**) and α-HBDH (**G**) using colorimetric assay kits with a cobas c311 analyzer in these groups (*n* = 3 to 5 per group); (**H**,**I**) Measurements of serum cTNI (**H**) and NT-proBNP (**I**) using ELISA kits in these groups (*n* = 5 to 9 per group). Data are shown as the mean ± s.d. Statistical analyses in (**A**–**D**,**F**–**I**) were performed with one-way ANOVA with Dunnett’s multiple comparisons test.

As TMP is widely used in treating ischemic cardiovascular diseases, we speculated that TMP might alleviate the cardiac dysfunction induced by chronic hypobaric hypoxia exposure. To test our hypothesis, we treated mice with saline (Hypoxia) or TMP at different doses of 5 mg/kg (TMP5), 20 mg/kg (TMP20), and 100 mg/kg (TMP100) by means of daily gavage for 4 weeks during hypobaric hypoxia exposure. The normoxic control adult mice treated with saline (Normoxia) were maintained in a sea-level environment for a similar period. Enalapril was chosen as the positive control, as enalapril is reported to ameliorate hypobaric hypoxia-induced right ventricular hypertrophy and fibrosis [36]. The mice of the Ena group were treated with 20 mg/kg enalapril by means of daily gavage for 4 weeks during hypobaric hypoxia exposure. Echocardiographic parameters and serum biochemicals were detected following the 4-week treatment. The average RVFAC of the Ena (0.46 ± 0.11), TMP5 (0.50 ± 0.08), TMP20 (0.48 ± 0.10), or TMP100 (0.48 ± 0.11) groups was significantly higher than that of the Hypoxia group (0.26 ± 0.095), as determined by one-way ANOVA with Dunnett’s multiple comparisons test (*p* < 0.05) (Figure 2A). Thus, the TMP treatment improved the right ventricular systolic function under hypobaric hypoxia. We further detected TAPSE on an apical four-chamber view via echocardiography. Compared with the Hypoxia group (0.72 ± 0.18 mm), the average TAPSE of the TMP5 (1.07 ± 0.16 mm) or the TMP20 (1.05 ± 0.14 mm) groups was significantly elevated (Dunnett’s test, *p* < 0.01), indicating that TMP treatment improved TAPSE under hypobaric hypoxia (Figure 2B). Thus, TMP improves right ventricular systolic function.

Then, we measured the end-systolic right ventricular internal diameter (RVIDs) and RVIDd, indicators of the size of the right ventricular cavity, on the parasternal short-axis section view via echocardiography. The average RVIDs of the Ena (1.14 ± 0.19 mm), TMP5 (1.01 ± 0.34 mm), TMP20 (0.98 ± 0.31 mm), or TMP100 (0.87 ± 0.15 mm) groups was shorter than that of the Hypoxia group (1.51 ± 0.22 mm), as determined by one-way ANOVA with Dunnett’s multiple comparisons test (*p* < 0.05) (Figure 2C). Moreover, the average RVIDd of the TMP5 (1.46 ± 0.16 mm), TMP20 (1.59 ± 0.31 mm), or TMP100 (1.42 ± 0.44 mm) groups was shorter than that of the Hypoxia group (2.07 ± 0.29 mm), as determined by one-way ANOVA with Dunnett’s multiple comparisons test (*p* < 0.05) (Figure 2D,E). These results demonstrate TMP alleviates right ventricular enlargement.

We further detected the content of myocardial injury markers creatine kinase-MB (CK-MB), α-hydroxybutyrate dehydrogenase (α-HBDH), and cardiac troponin I (cTnI) in mouse serum. Although the average CK-MB content of the Hypoxia group did not significantly increase compared with that of the Normoxia group (Dunnett’s test, *p* = 0.1074), the enalapril (243.6 ± 78.38 U/L), 5 mg/kg TMP (229.3 ± 127.10 U/L), 20 mg/kg TMP (131.17 ± 90.68 U/L), or 100 mg/kg TMP (146.3 ± 55.57 U/L) treatments reduced average CK-MB content compared to saline treatment (503 ± 134.55 U/L) in hypobaric hypoxic mice (Dunnett’s test, *p* < 0.01) (Figure 2F). We further analyzed the α-HBDH content of these groups. The average α-HBDH content of the Hypoxia group (681.25 ± 275.81 U/L) increased compared with that of the Normoxia group (355 ± 78.90 U/L), as determined by Dunnett’s test (*p* < 0.01), indicating that hypobaric hypoxia resulted in myocardial injury (Figure 2G). The Ena (204 ± 72.15 U/L), TMP5 (169 ± 57.38 U/L), TMP20 (150 ± 103.32 U/L), or TMP100 (105 ± 30.62 U/L) group had reduced serum α-HBDH content compared to the Hypoxia group (681.25 ± 275.81 U/L) (Dunnett’s test, *p* < 0.01), demonstrating that TMP treatment alleviated hypobaric hypoxia-induced myocardial injury (Figure 2G). We next measured serum cTnI content using an enzyme-linked immunosorbent assay (ELISA). Similarly, the average cTnI content of the Hypoxia group (276.30 ± 10.69 pg/mL) increased compared with that of the Normoxia group (178.89 ± 16.42 pg/mL) (Dunnett’s test, *p* < 0.001). The enalapril (228.73 ± 11.62 pg/mL), 5mg/kg TMP (253.11 ± 9.30 pg/mL), 20 mg/kg TMP (243.83 ± 5.38 pg/mL), or 100 mg/kg TMP (223.13 ± 7.30 pg/mL) treatments reduced average cTnI content compared to saline treatment (276.30 ± 10.69 pg/mL) in hypobaric hypoxic mice (Dunnett’s test, *p* < 0.001) (Figure 2H). These results demonstrated TMP reduces hypobaric hypoxia-induced myocardial injury. Hypoxia results in the release of brain natriuretic peptides (BNPs) and amino-terminal pro-brain natriuretic peptides (NT-proBNP), which are indicators of cardiac function. Thus, we next determined NT-proBNP levels in these groups. The average NT-proBNP content of Hypoxia group (44.76 ± 1.63 pg/mL) was higher than that of Normoxia group (30.10 ± 1.88 pg/mL ), as determined by Dunnett’s test (*p* < 0.001), demonstrating that hypobaric hypoxia impaired cardiac function. The Ena (37.38 ± 1.17 pg/mL), TMP20 (40.34 ± 0.77 pg/mL), or TMP100 (37.41 ± 1.70 pg/mL) groups had reduced NT-proBNP content compared to the Hypoxia group (44.76 ± 1.63 pg/mL) (Dunnett’s test, *p* < 0.001), indicating TMP treatment ameliorated hypobaric hypoxia-induced cardiac dysfunction (Figure 2I).

Collectively, these results provide evidence that TMP improves RV function, relieves RV enlargement, and alleviates myocardial injury in hypobaric hypoxic mice.

### 2.3. TMP Alleviates Hypobaric Hypoxia-Induced Cardiac Hypertrophy

**Figure 3:**
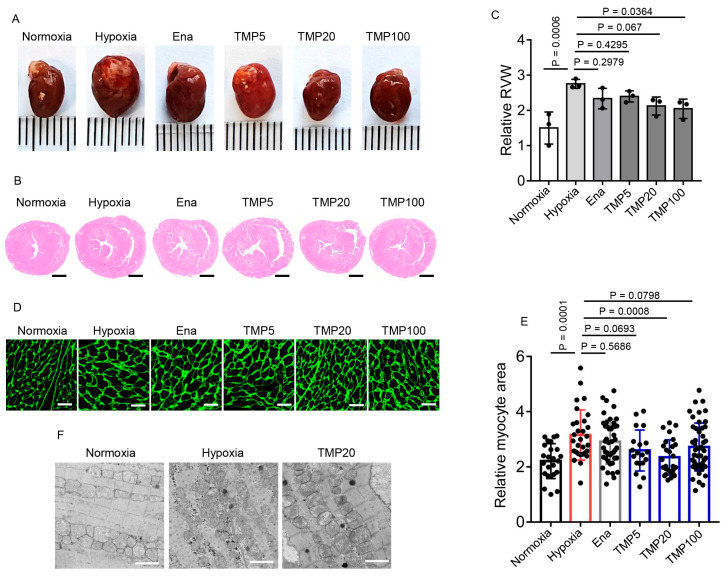
TMP alleviates hypobaric hypoxia-induced cardiac hypertrophy. Mice were treated with saline, enalapril (20 mg/kg), or TMP at different doses (5, 20, or 100 mg/kg) by means of daily gavage for 4 weeks during hypobaric hypoxia exposure, named a Hypoxia group, Ena group, TMP5 group, TMP20 group, and TMP100 group, respectively. Normoxic mice treated with saline for 4 weeks served as a control, named a Normoxia group. The following data were collected after the 4-week experiment: (**A**) Representative photographs of hearts derived from these groups; (**B**) Representative H&E staining of the myocardial cross sections derived from these groups. Scale bar: 1 mm; (**C**) Statistical analysis of the relative right ventricular wall thickness as mentioned in (**B**) (*n* = 3 per group); (**D**) Representative images of wheat germ agglutinin (WGA) staining in myocardial tissues derived from these groups. Scale bar: 10 μm; (**E**) Quantification of the relative cardiomyocyte cross-sectional area as mentioned in (**D**) (*n* = 3 mice per group); (**F**) Representative electron microscope images of mitochondria in the myocardial tissues derived from the Normoxia, Hypoxia, and TMP20 groups. Scale bar: 2 μm. Data are shown as the mean ± s.d. Statistical analyses in (**C**,**E**) were performed with one-way ANOVA with Dunnett’s multiple comparisons test.

We further evaluated whether TMP reversed the cardiac morphological changes induced by hypobaric hypoxia. Mice from the Normoxia, Hypoxia, Ena, TMP5, TMP20, and TMP100 groups described above were euthanized after a 4-week experiment, and the hearts were collected (Figure 3A). To evaluate the effect of TMP treatment on hypobaric hypoxia-induced RV hypertrophy, we conducted hematoxylin–eosin staining on cross-sections of heart tissues and measured RV wall thickness by ImageJ 1.52a software. The average RV wall thickness of the Hypoxia group (2.75 ± 0.13) significantly increased compared to that of the Normoxia group (1.50 ± 0.46), as determined by one-way ANOVA with Dunnett’s test (Dunnett’s test, *p* < 0.001), indicating hypobaric hypoxia-induced RV hypertrophy (Figure 3B,C). Furthermore, compared to the Hypoxia group (2.75 ± 0.13), the average RV wall thickness of the TMP100 group (2.05 ± 0.27) was significantly reduced, although the average RV wall thickness of the Ena (2.33 ± 0.30), TMP5 (2.40 ± 0.16), and TMP20 (2.13 ± 0.25) groups had no significant changes (Dunnett’s test, *p* > 0.05), indicating that TMP alleviated hypobaric hypoxia-induced RV hypertrophy at a high dose. We next detected the cardiomyocyte cross-sectional area by wheat germ agglutinin (WGA) staining on cross sections of the heart tissues. The cardiomyocyte cross-sectional area was calculated by tracing the outlines of cardiomyocytes using Adobe Photoshop CS6 software. Compared to the Hypoxia group (3.15 ± 0.91), the TMP20 group (2.34 ± 0.64) had significantly reduced average cardiomyocyte cross-sectional area, as determined by one-way ANOVA with Dunnett’s test (Dunnett’s test, *p* < 0.001) (Figure 3D,E), although Ena (2.91 ± 0.81), TMP5 (2.59 ± 0.74), and TMP100 (2.72 ± 0.86) groups had no significant changes (Dunnett’s test, *p* > 0.05) (Figure 3D,E). Furthermore, hypobaric hypoxia rendered fragmented mitochondria, which was reversed by 20 mg/kg TMP treatment, as observed under electron microscopy (Figure 3F). Collectively, these results demonstrate that TMP alleviates hypobaric hypoxia-induced cardiac hypertrophy at high doses.

### 2.4. TMP Enhances Myocardial Contraction

**Figure 4:**
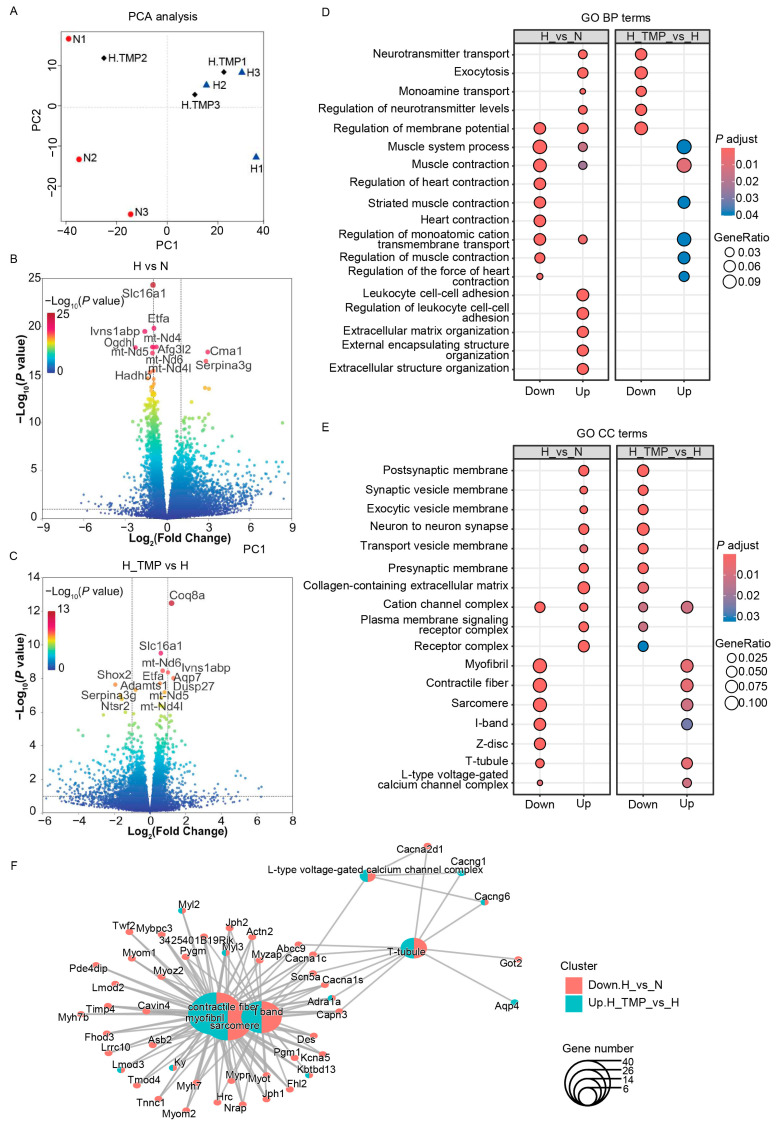
TMP enhances myocardial contraction. Hypobaric hypoxic mice were treated with saline (H, *n* = 3) or TMP at 20 mg/kg (H_TMP, *n* = 3) daily for 4 weeks during hypobaric hypoxia exposure. Normoxic mice were treated with saline for 4 weeks (N, *n* = 3). (**A**) Unsupervised classification using principal component analysis (PCA) on the cardiac global gene expression profiles generated via bulk RNA sequencing. (**B**) Volcano plot of differentially expressed genes for the hypobaric hypoxia group versus the normoxia group (H vs. N) (*p*-value < 0.05, |log2(fold change)| > 1). (**C**) Volcano plot of differentially expressed genes for the TMP-treated hypobaric hypoxia group versus the control solvent-treated hypobaric hypoxia group (H_TMP vs. H) (*p*-value < 0.05, |log2(fold change)| > 1). (**D**) GO biological process (BP) enrichment of differentially expressed genes in H vs. N and H_TMP vs. H. (**E**) GO cellular component (CC) enrichment of differentially expressed genes in H vs. N and H_TMP vs. H. (**F**) Network diagram of GO CC enriched terms and linked genes. Red indicates downregulated genes in the H vs. N cluster, and blue indicates upregulated genes in the H_TMP vs. H cluster. The point size corresponds to the gene number.

To investigate the molecular mechanisms underlying the effects of TMP on hypobaric hypoxia-induced cardiac dysfunction, we conducted RNA sequencing. The mice were treated with saline (H) or 20 mg/kg TMP (H_TMP) by means of daily gavage for 4 weeks during hypobaric hypoxia exposure. The normoxic control adult mice treated with saline (N) were maintained in a sea-level environment for a similar period. The mice were euthanized after a 4-week experiment, and the hearts were collected for RNA sequencing analysis. A sample classification by means of principal component analysis (PCA) of all the detected genes showed that the three groups were separate from each other (Figure 4A). To further characterize the differentially expressed genes from the RNA sequencing data, we set thresholds of *p* < 0.05 and |log2FC| > 1 using the DESeq2 R package (1.42.1). In total, 1563 upregulated genes and 401 downregulated genes were identified after hypobaric hypoxia exposure (Figure 4B). TMP treatment resulted in 81 upregulated genes and 315 downregulated genes in hypobaric hypoxic mice (Figure 4C). A Gene Ontology (GO) analysis of biological processes indicated that compared with the normoxia (N) group, genes related to heart contraction and muscle contraction were sharply downregulated in the hypobaric hypoxia (H) group, whereas the TMP intervention (H_TMP) significantly reversed these processes (Figure 4D). A further GO analysis of cellular components showed that hypobaric hypoxia impaired the myofibrils, contractile fibers, sarcomeres, I-bands, Z-discs, and T-tubules of cardiac myocytes; however, these impairments were reversed by TMP treatment (Figure 4E,F). These results indicate that TMP enhances heart contraction and maintains the myocardial fiber structure, which explains the improvement in cardiac function with TMP treatment under hypobaric hypoxia.

### 2.5. TMP Inhibits Hypobaric Hypoxia-Induced CaMKII Activation in Mouse Heart

**Figure 5:**
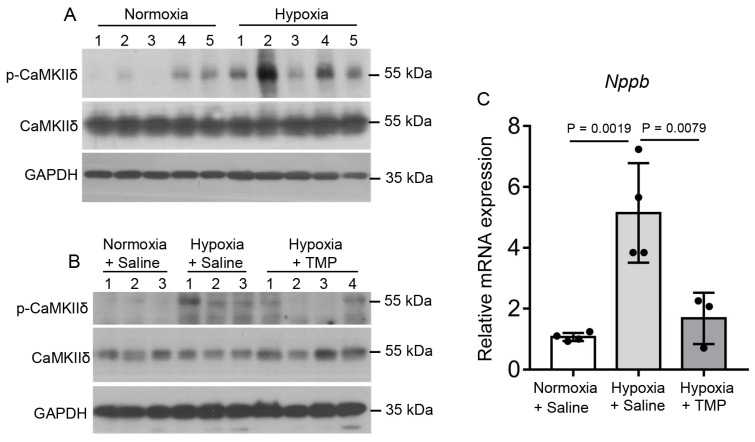
TMP inhibits hypobaric hypoxia-induced CaMKII activation in mouse hearts. (**A**) Mice were exposed to normoxia or hypobaric hypoxia for 4 weeks, after which they were euthanized, and the expressions of p-CaMKIIδ, CaMKIIδ, and GAPDH in heart tissues were detected by Western blot analysis (*n* = 5 mice per group). (**B**) Mice were treated with saline (Hypoxia + Saline) or 20 mg/kg TMP (Hypoxia + TMP) during 4-week hypobaric hypoxia exposure. Mice treated with saline during 4-week normoxia (Normoxia + Saline) served as a negative control. The expressions of p-CaMKIIδ, CaMKIIδ, and GAPDH in heart tissues from these mice were detected by Western blot analysis (*n* = 3 or 4 mice per group). (**C**) Relative mRNA levels of *Nppb* in heart tissues from mice mentioned in (**B**) (*n* = 3 to 4 mice per group). Data are shown as the mean ± s.d. Statistical analyses in (**C**) were performed with one-way ANOVA with Tukey post hoc tests.

The serine/threonine protein kinase CaMKII is activated by Ca^2+^ and has been shown to be involved in several pathological cardiac conditions. CaMKII activation mediates the hypoxia-induced increase in the late sodium current [35]. Thus, we detected the phosphorylation levels of CaMKIIδ in the heart tissues of normoxic and hypobaric hypoxic mice. Indeed, compared to 4-week normoxic exposure, 4-week hypobaric hypoxic exposure increased the phosphorylation levels of CaMKIIδ in the heart, indicating that CaMKIIδ was activated by hypobaric hypoxia (Figure 5A). Daily TMP treatment at 20 mg/kg during 4-week hypobaric hypoxic exposure (Hypoxia + TMP) significantly decreased the CaMKIIδ phosphorylation levels in heart tissues compared to saline treatment during hypobaric hypoxic exposure (Hypoxia + Saline) (Figure 5B), demonstrating that TMP inhibits hypobaric hypoxia-induced CaMKIIδ activation. As CaMKII activation facilitated hypertrophy-related gene transcription [31], we further examined the levels of the hypertrophic gene *Nppb* in the heart tissues by means of qPCR. Compared to 4-week normoxic exposure (1.07 ± 0.13), 4-week hypobaric hypoxic exposure (5.15 ± 1.64) increased the *Nppb* transcription levels in the heart (Tukey’s test, *p* < 0.01), indicating that hypobaric hypoxia increased hypertrophy-related gene transcription (Figure 5C). Daily TMP treatment at 20 mg/kg during 4-week hypobaric hypoxia exposure (1.68 ± 0.84) significantly decreased the Nppb transcription levels compared to the saline treatment during hypobaric hypoxia exposure (5.15 ± 1.64), as determined by one-way ANOVA with Tukey’s multiple comparisons test (Figure 5C). These results demonstrate that TMP decreases hypobaric hypoxia-induced CaMKII activation in mice hearts.

### 2.6. TMP Improves Cardiac Function Through Inhibiting CaMKII Activation in Hypobaric Hypoxic Mice

As CaMKII is a major determinant of cardiac function, we next examined whether TMP improved cardiac function in a CaMKII-dependent manner. KN93 is a cell-permeable, reversible, and competitive inhibitor of CaMKII [37]. Thus, we utilized KN93 to study the role of CaMKII in hypobaric hypoxic mouse hearts and determine whether TMP exerted an effect through CaMKII. The mice were treated with saline (Ctrl), 10 mg/kg TMP (TMP), 1.5 mg/kg KN93 (KN93), or both 10 mg/kg TMP and 1.5 mg/kg KN93 (TMP_KN93) daily during normoxia or hypobaric hypoxia exposure for 4 weeks. The TMP solution was given by gavage, while the KN93 solution was given by intraperitoneal injection. Saline was given to mice to ensure that each mouse was given the same amount of solvent.

**Figure 6:**
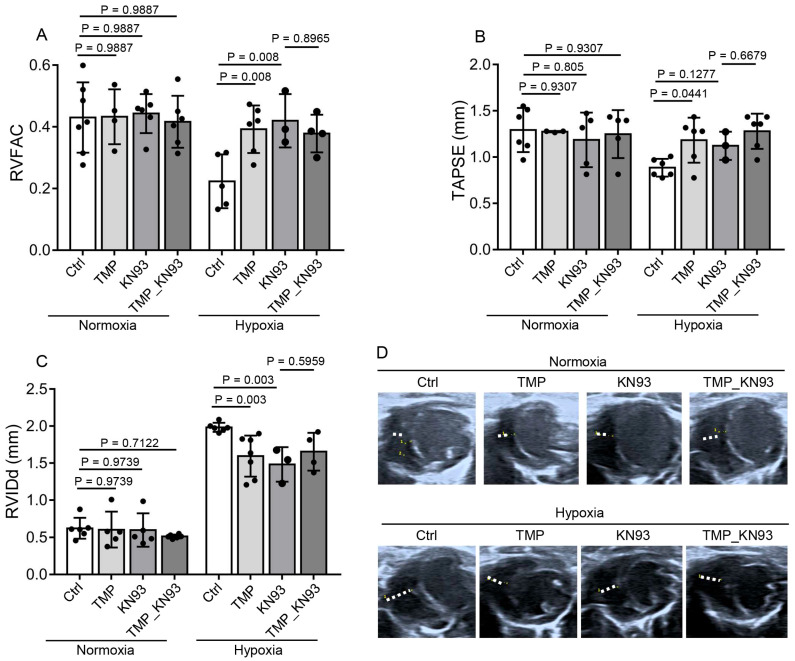
TMP improves cardiac function by inhibiting CaMKII activation in hypobaric hypoxic mice. The mice were treated with saline (Ctrl), 10 mg/kg TMP (TMP), 1.5 mg/kg KN93 (KN93), or both 10 mg/kg TMP and 1.5 mg/kg KN93 (TMP_KN93) daily during normoxia or hypobaric hypoxia exposure for 4 weeks. The following data were collected: (**A**–**C**) Measurements of the RVFAC (**A**), TAPSE (**B**), and RVIDd (**C**) by means of echocardiography in the Ctrl, TMP, KN93, and TMP_KN93 groups after 4-week normoxia or hypobaric hypoxia exposure (*n* = 3 to 7 per group); (**D**) Representative images of RVIDd measurement on a parasternal short-axis section view in these groups described in (**C**); Data are shown as the mean ± s.d. Statistical analyses in (**A**–**C**) were performed with two-way ANOVA with Holm–Sidak’s multiple comparisons test.

Under normoxia, TMP or KN93 had no effect on RVFAC, as determined by two-way ANOVA with Holm–Sidak’s multiple comparisons test (*p* > 0.05) (Figure 6A). Under hypobaric hypoxia, the TMP group (0.39 ± 0.08) or the KN93 group (0.42 ± 0.09) had increased average RVFAC compared to the Ctrl group (0.22 ± 0.09), as determined by two-way ANOVA with Holm–Sidak’s multiple comparisons test (*p* < 0.01) (Figure 6A), demonstrating that CaMKII inhibition improves right ventricular systolic function under hypobaric hypoxia. Moreover, under hypobaric hypoxia, compared to the KN93 group (0.42 ± 0.09), the TMP_KN93 group (0.38 ± 0.06) had similar average RVFAC (Holm–Sidak’s test, *p* > 0.05), demonstrating that TMP treatment did not further increase the RVFAC when utilized together with KN93 compared to KN93 alone; thus, TMP improves right ventricular systolic function in a CaMKII-dependent manner (Figure 6A). We further measured TAPSE in these groups. Under normoxia, TMP or KN93 had no effect on TAPSE, as determined by two-way ANOVA with Holm–Sidak’s multiple comparisons test (*p* > 0.05) (Figure 6B). Under hypobaric hypoxia, the TMP group (1.18 ± 0.24 mm) had an increased average TAPSE compared to the Ctrl group (0.89 ± 0.10 mm) (Holm–Sidak’s test, *p* < 0.05). However, KN93 at 1.5 mg/kg (1.12 ± 0.15 mm) had no obvious effect on TAPSE compared to saline (0.89 ± 0.10 mm) under hypobaric hypoxia (Holm–Sidak’s test, *p* > 0.05), which might be caused by insufficient KN93 dose (Figure 6B). We next determined the RVIDd in these groups. Under normoxia, TMP or KN93 had no effect on RVIDd, as determined by two-way ANOVA with Holm–Sidak’s multiple comparisons test (*p* > 0.05) (Figure 6C). Under hypobaric hypoxia, the TMP group (1.59 ± 0.28 mm) or the KN93 group (1.48 ± 0.23 mm) had decreased average RVIDd compared to the Ctrl group (1.98 ± 0.06 mm), as determined by two-way ANOVA with Holm–Sidak’s multiple comparisons test (*p* < 0.01) (Figure 6C,D), demonstrating that CaMKII inhibition alleviated right ventricular enlargement under hypobaric hypoxia. Moreover, under hypobaric hypoxia, compared to the KN93 group (1.48 ± 0.23 mm), the TMP_KN93 group (1.65 ± 0.26 mm) had similar average RVIDd (Holm–Sidak’s test, *p* > 0.05), demonstrating TMP treatment did not further decrease the RVIDd when utilized together with KN93 compared to KN93 alone; thus, TMP relieves right ventricular enlargement in a CaMKII-dependent manner (Figure 6C,D). Taken together, these results demonstrate that TMP improves cardiac function through inhibiting CaMKII activation in hypobaric hypoxic mice.

### 2.7. TMP Alleviates Cardiac Hypertrophy Through Inhibiting CaMKII Activation in Hypobaric Hypoxic Mice

**Figure 7:**
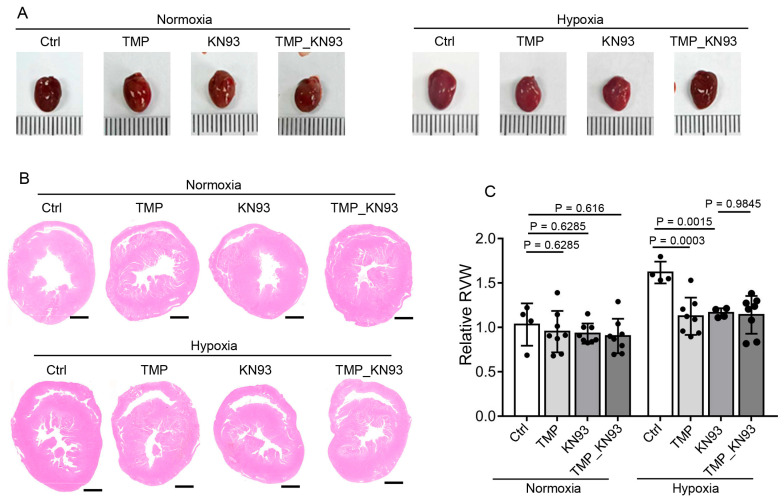
TMP alleviates cardiac hypertrophy by inhibiting CaMKII activation in hypobaric hypoxic mice. Mice were treated with saline (Ctrl), 10 mg/kg TMP (TMP), 1.5 mg/kg KN93 (KN93), or both 10 mg/kg TMP and 1.5 mg/kg KN93 (TMP_KN93) daily during normoxia or hypobaric hypoxia exposure for 4 weeks. The following data were collected after 4-week experiment: (**A**) Representative photographs of hearts derived from these groups; (**B**) Representative H&E staining of the myocardial cross sections derived from these groups. Scale bar: 1 mm; (**C**) Statistical analysis of the relative right ventricular wall thickness (RVW) as mentioned in (**B**) (*n* = 4 to 8 per group). Data are shown as the mean ± s.d. Statistical analyses in (**C**) were performed with two-way ANOVA with Holm–Sidak’s multiple comparisons test.

We further examined whether TMP alleviates cardiac hypertrophy in a CaMKII-dependent manner. Mice from the Ctrl, TMP, KN93, and TMP_KN93 groups described above were euthanized after 4-week normoxic or hypobaric hypoxic exposure, and the hearts were collected (Figure 7A). We further conducted hematoxylin–eosin staining on cross-sections of heart tissues and measured RV wall thickness by ImageJ software (Figure 7B). Under normoxia, TMP or KN93 had no effect on RV wall thickness, as determined by two-way ANOVA with Holm–Sidak’s multiple comparisons test (*p* > 0.05) (Figure 7C). Under hypobaric hypoxia, the TMP group (1.13 ± 0.21) or the KN93 group (1.16 ± 0.05) had decreased average RV wall thickness compared to the Ctrl group (1.62 ± 0.12), as determined by two-way ANOVA with Holm–Sidak’s multiple comparisons test (*p* < 0.01) (Figure 7C), indicating that CaMKII inhibition alleviated hypobaric hypoxia-induced RV hypertrophy. Moreover, under hypobaric hypoxia, compared to the KN93 group (1.16 ± 0.05), the TMP_KN93 group (1.14 ± 0.21) had similar average RV wall thickness (Holm–Sidak’s test, *p* > 0.05) (Figure 7C), demonstrating that TMP treatment did not further decrease the RV wall thickness when utilized together with KN93 compared to KN93 alone; thus, TMP alleviates hypobaric hypoxia-induced cardiac hypertrophy through inhibiting CaMKII activation.

## 3. Discussion

TMP exerts therapeutic effects against a variety of cardiovascular diseases, such as atherosclerosis, myocardial ischemia, arrhythmia, and dilated cardiomyopathy [14,38,39,40]. The pharmacological effects of TMP include antioxidant, anti-inflammatory, anti-platelet, and anti-apoptotic effects [41,42,43]. Nevertheless, there have been no previous clues indicative of the possible effect of TMP on high-altitude-induced cardiac dysfunction. In this study, we observed the benefits of TMP treatment to mouse hearts under simulated high-altitude hypobaric hypoxia, with protective effects against RV dysfunction, RV dilation, and cardiac hypertrophy.

Excessive CaMKII activation can contribute to dilated cardiomyopathy, myocardial hypertrophy, heart failure, myocardial ischemia, and arrhythmia through a variety of processes. The inhibition of CaMKII results in enhanced contractility in the isolated myocardium of a failing heart. The mechanism involves restoring the Ca^2+^ load in the sarcoplasmic reticulum [28]. However, the role of CaMKII in hypobaric hypoxia-induced cardiac dysfunction remains elusive. In this study, we found that CaMKII phosphorylation was increased in mouse hearts after chronic hypobaric hypoxia exposure, demonstrating that CaMKII is activated by such exposure. Although we did not further investigate the underlying mechanism, we speculate that chronic β-adrenergic activation might contribute to CaMKII phosphorylation as high-altitude exposure results in activation of the adrenergic system, which increases intracellular Ca^2+^ levels, leading to calcified calmodulin (Ca^2+^/CaM) binding to CaMKII [25,44,45]. By utilizing the CaMKII inhibitor KN93, we found that CaMKII inhibition protected mice from decreased contractile function and cardiac hypertrophy under chronic hypobaric hypoxia. Thus, we uncovered a function of CaMKII in controlling cardiac function and hypertrophy under chronic hypobaric hypoxia, and we suggest CaMKII as a potential target for cardiac dysfunction induced by high-altitude exposure.

As a calcium antagonist, TMP prevents intracellular calcium overload by blocking the entry of extracellular calcium or by inhibiting the release of intracellular calcium stores [46,47,48]. As intracellular calcium is a key activator of CaMKII, we speculate that TMP might inhibit CaMKII activation by suppressing intracellular calcium overload. However, the exact mechanism still needs further investigation.

Cardiac muscle cells are enriched in mitochondria (35% of their cell volume) due to the high energy demands of the heart. A majority of the ATP generated through oxidative phosphorylation is required to sustain the Ca^2+^-dependent contraction of cardiomyocytes [49]. Consequently, the maintenance of mitochondrial homeostasis is crucial for proper heart function. In this study, we found that chronic hypobaric hypoxia exposure dramatically altered the mitochondrial morphology in mouse hearts, characterized by a disordered arrangement and reduced mitochondrial size (Figure 3F), which may contribute to the impairment in cardiac function under hypobaric hypoxia. Indeed, previous studies indicated that chronic hypobaric hypoxia led to a decrease in the size of mitochondria and an increase in their number in adult rats, and it also modestly reduced electron transfer chain complex activity [50]. Moreover, hypoxia damages mitochondrial components, compromises cellular energy storage, and influences mitochondrial dynamics and the mitochondrial cell death pathways [51]. CaMKII activation has been reported to result in the opening of mitochondrial permeability transition pores (mPTPs) and increased reactive oxygen species production, indicating that activated CaMKII causes mitochondrial injury [52]. Thus, activated CaMKII may be one contributor to mitochondrial impairment in hypobaric hypoxic mouse hearts. However, future works should be performed to elucidate whether CaMKII–mitochondrion signaling exists and causes cardiac dysfunction in hypobaric hypoxic mouse hearts.

Overall, we propose a model in which TMP protects against hypobaric hypoxia-induced cardiac dysfunction by inhibiting CaMKII overactivation. TMP not only improves cardiac function but also alleviates myocardial injury and cardiac hypertrophy in hypobaric hypoxic mice. This discovery suggests that TMP is a promising drug candidate for treating cardiac dysfunction induced by high-altitude exposure.

## 4. Materials and Methods

### 4.1. Animals

The C57BL/6 J mice used in this study were bred in a pathogen-free environment. All the animal experiments adhered to the ethics of animal research. For the hypobaric hypoxia exposure, 6-week-old male mice were kept in a hypobaric chamber with the pressure at about 47 kPa (equivalent to the atmospheric pressure at 6000 m) for 4 weeks. For the TMP (Macklin, Shanghai, China, T819555) or enalapril (Macklin, E830566) treatments, mice were administered with TMP or enalapril dissolved in saline at the indicated dose daily by gavage during normoxic or hypobaric hypoxic exposure. For the KN93 (MCE, HY-15465) treatment, mice were intraperitoneally injected with KN93 dissolved in 0.5% DMSO at 1.5 mg/kg/day. The vehicle control groups were given an equivalent volume of saline or 0.5% DMSO.

### 4.2. Echocardiography

The mice were shaved with depilatory creams and then anesthetized with 3% isoflurane (RWD, R510-22-10) in a chamber. The anesthesia states were maintained with 1.5% isoflurane, administered through a mask. The mice were laid on a platform with all four limbs linked to electrocardiogram electrodes. The echocardiographs were detected using a VINNO 6LAB real-time ultrasound scanner. The echocardiograph at the apical four-chamber view was obtained by placing the probe at the apex of the mouse’s heart, with the probe’s mark point facing the mouse’s left forelimb. The outlines of the RV end-diastolic area and RV end-systolic area were traced at the apical four-chamber view, and the RVFACs were calculated using the following formula: (RV end-diastolic area—RV end-systolic area)/(RV end-diastolic area) × 100%. The TAPSE was obtained by placing the sampling line on the lateral tricuspid annulus and tracking the displacement of the tricuspid annulus from end-diastole to end-systole at the apical four-chamber view. The echocardiograph at the parasternal short-axis view was obtained by placing the probe in a parasternal position, with the probe’s mark pointing toward the 3 o’clock direction. The RVID was measured at an apical four-chamber view using Adobe Photoshop software. The CO was measured and calculated using methods described in the literature [53].

### 4.3. Hemodynamic Measurements

Mice were given 2–3% isoflurane gas and fixed on a mouse plate. A median incision was made after neck skin preparation, and approximately 0.6 cm of the external jugular vein was freed. The upper vein was ligated with surgical thread. A polyethylene catheter was connected to a pressure sensor, which was then connected to a Data Acquisition System (PowerLab, AD instruments, New South Wales, Australia). A syringe with heparin saline was connected to the system using a tee tube to flush the tube and prevent coagulation. A “V” incision was gently cut into the external jugular vein, and the catheter tip was guided along the “V” incision into the vein. The catheter was fixed by means of ligation of the lower vein. The catheter was advanced into the right ventricle to obtain the right ventricular end-diastolic pressure (RVEDP) and the right ventricular systolic pressure (RVSP).

### 4.4. RNA Sequencing and Data Analysis

The transcriptomic alterations were detected by means of RNA sequencing. Mice were kept under normoxic or hypobaric hypoxic conditions and gavaged with control solvent or TMP (20 mg/kg) daily for 4 weeks. Heart tissues from the mice were then collected for total RNA extraction using TRIzol reagents (15596026, Invitrogen, Thermo Fisher Scientific, Waltham, MA, USA). Sequencing library preparation and RNA sequencing were performed using Illumina platforms at Novogene Technology Company (Beijing, China). After data quality control using Fastp (version 0.23.1), HISAT2 (version 0.1.6-beta) was used to map clean reads to the mouse genome. The differentially expressed genes (DEGs) were selected with *p* < 0.05 and |log2FC| > 1 as the thresholds using DEseq2 (version 1.5.3). GO enrichment analyses of the biological processes and cellular components of the DEGs were performed using the R package clusterProfiler (version 4.10.0).

### 4.5. Measurement of Myocardial Injury Markers

The mice were anesthetized, and their blood was collected. The serum was retained after blood centrifugation. The CK-MB and α-HBDH contents in serum were detected using a CK-MB colorimetric assay kit (07190808190, Roche, Basel, Switzerland) and an α-HBDH colorimetric assay kit (06750036190, Roche, Basel, Switzerland) with cobas c311 analyzer (Roche, Basel, Switzerland), following the established protocols. The concentrations of NT-proBNP (MM-0558M2, MEIMIAN, Yancheng, Jiangsu, China) and cTNI (MM-46632M1, MEIMIAN, Jiangsu, China) were detected using enzyme-linked immunosorbent assay (ELISA) kits. Briefly, 10 μL of serum was added to the coated wells of the ELISA plate, followed by incubation at 37 °C for 30 min. The wells were washed five times, and HRP-conjugated enzyme reagent was added. After incubation at 37 °C for another 30 min, the wells were washed five times, and chromogenic solutions A and B were added. The plate was incubated at 37 °C for 10 min for color development. The reaction was stopped by adding the stop solution, and the optical density (OD) values were measured on a microplate reader (VICTOR X, PerkinElmer, Waltham, MA, USA) within 15 min.

### 4.6. Histology

Heart tissues were fixed with 4% formaldehyde for two days, and then the tissues were dehydrated by alcohol at 70%, 80%, 90%, and 100% concentrations for 2 min each. The tissues were then placed in xylene for 1 h and then embedded in paraffin. The embedded tissues were sectioned using a slicer (RM2125 RTS, Leica, Wetzlar, Germany) to obtain 4 μm slices. The thin slices were floated on a warm water bath to flatten and then picked up on a glass microscope slide. The slides were dried and immersed in xylene for 10 min to dewax. Then, the slides were rehydrated by graded alcohols at 70%, 80%, 90%, and 100% concentrations for 2 min each. Then, the slides were stained with hematoxylin for 2 min and rinsed with water. The slides were then stained with eosin for 5 min and rinsed with water. Then, the slides were dehydrated through a series of alcohol solutions (80%, 90%, and 100%) and cleared with xylene. Then, the slides were sealed by coverslips. The slides were scanned by a scanner (Pannoramic DIMI, 3DHISTECH, Budapest, Hungary) and viewed by CaseViewer2.4 software. The relative right ventricular wall thickness was measured using ImageJ software. The average value of the right ventricular wall thickness measured at ten different locations is taken as the right ventricular wall thickness for each sample.

### 4.7. Wheat Germ Agglutinin (WGA) Analyses

Heart tissues were collected and fixed with 4% formaldehyde for two days, and then, the tissues were dehydrated through a series of graded alcohol solutions (70%, 80%, 90%, and 100% ethanol) to remove water. The tissues were then placed in xylene and embedded in paraffin. The embedded tissues were sectioned using a slicer (Leica, RM2125 RTS). The slides were dewaxed and dehydrated through graded alcohols. Then, the slides were placed in the citrate solution and heated for antigen retrieval. Then, the slides were washed twice with phosphate buffer. A stock solution of iF488-wheat germ agglutinin (G1730, Servicebio, Wuhan, China) was diluted to 5 µg/mL by PBS, and 50 μL of diluted solution was added to each slide. Then, the slides were protected from light and incubated for one hour at room temperature. Then, the sections were rinsed three times with phosphate buffer and mounted. The sections were visualized using a microscope (Ti-E, Nikon, Tokyo, Japan). The cross-sectional area of the cardiomyocytes was calculated by tracing the outlines of cardiomyocytes using Adobe Photoshop software.

### 4.8. Transmission Electron Microscopy

Heart tissues from the mice were sectioned into small pieces (1–2 mm^3^) and then fixed in a solution of 2.5% glutaraldehyde. Following this, the samples were incubated for 2 h with 1% osmium tetroxide. The samples were dehydrated through a graded series of acetone to remove water. Then, the samples were infiltrated with a mixture of resin and acetone, after which the samples were embedded in resin and sectioned using a Leica UC Enuity to obtain slices (70–100 nm). The slices were stained with lead citrate solution and a 50% ethanol-saturated solution of uranyl acetate for 15 min each and then observed under a transmission electron microscope (HT7800, Hitachi, Tokyo, Japan).

### 4.9. Western Blots

Heart tissues were weighed, and 100 mg of tissue was added with 1 mL of RIPA lysis buffer (P0013B, Beyotime, Shanghai, China). Then, the tissues were ground into tissue homogenate using a homogenizer (AM100, Ants scientific instruments, Beijing, China). The tissue homogenate was centrifuged, and the supernatant was collected for Western blot analyses. Then, the tissue lysates were mixed with loading buffer (Servicebio, G2013) and denatured by boiling for 10 min. The samples were loaded onto SDS–PAGE gels to separate the proteins at 100 V, after which the proteins on the gels were transferred to PVDF membranes at 90 mA, 3 h. After blocking with 5% skim milk for 1 h at room temperature, the membranes were incubated with primary antibodies targeting the following proteins for 3 h at room temperature: CaMKIIδ (20667-1-AP, Proteintech, Wuhan, China) at 1:5000 dilution; p-CaMKIIδ (YP0781, Immunoway, Plano, TX, USA) at 1:500 dilution; and GAPDH (60004-1-Ig, Proteintech, Wuhan, China) at 1:5000 dilution. Then, the membranes were incubated with HRP-conjugated secondary antibodies (ZB-2301, ZSGB-BIO, Chengdu, China) at 1:5000 dilution and detected using an enhanced chemiluminescence (ECL) system (SuperSignal™ West Pico PLUS, Thermo Scientific, Waltham, MA, USA) according to the manual. The original Western Blots can be found in Supplementary Materials.

### 4.10. Reverse Transcription Quantitative PCR (RT-qPCR)

A quantity of 50 mg of heart tissue was lysed with 1 mL of TRIzol reagent on ice. Every 1 mL of lysate was added with 0.2 mL of chloroform. The mixtures were centrifuged, and the aqueous phase was retained. The RNA was precipitated by adding an equal amount of isopropanol to the retained aqueous phase. Oligo (dT) primers that specifically bind to the Poly (A) tail of mRNA were used to generate cDNA via PCR using a reverse transcription assay kit (A230, Genstar, Beijing, China) with the following PCR procedure: 42 °C, 15 min; 85 °C, 5 s; 4 °C, 4 min. The commercial qPCR super mix (AQ631, TransGen Biotech, Beijing, China) was used to perform quantitative PCR according to the recommended procedure on a quantitative PCR system (CFX96, Biorad, Hercules, CA, USA) with the following procedure: hold stage (95 °C, 3 min); PCR stage (40 cycles: 95 °C, 10 s; 56 °C, 30 s; 72 °C, 20 s); melt curve stage (95 °C, 10 s; 65 °C, 1 min; 65 °C to 95 °C, increment 0.5 °C/s). The relative expression of *mNppb* was calculated by means of the ΔΔCt method and was normalized to that of *mGapdh*. RT-qPCR primers: *mNppb*: F: GAGGTCACTCCTATCCTCTGG, R: GCCATTTCCTCCGACTTTTCTC; *mGapdh*: F: AGGTCGGTGTGAACGGATTTG; R: TGTAGACCATGTAGTTGAGGTCA.

### 4.11. Statistical Analysis

The two-tailed Student’s *t*-test was used to determine the significance of differences between the two groups. One-way ANOVA was employed to compare three or more groups. Two-way ANOVA was used to compare three or more groups with two different factors. GraphPad Prism 7 was used to analyze the data and draw the figures. Data are shown as the mean ± s.d. Error bars represent the standard deviation (s.d.) of the mean. *p* < 0.05 was considered to indicate a statistically significant difference.

## Data Availability

The data supporting this study’s findings are available upon request to the corresponding author.

## Supplementary Materials

The following supporting information can be downloaded at https://www.mdpi.com/article/10.3390/ijms26010054/s1.

## References

[1] Davies S.W., Wedzicha J.A. (1993). Hypoxia and the heart. Br. Heart J..

[2] Fukuda T., Maegawa T., Matsumoto A., Komatsu Y., Nakajima T., Nagai R., Kawahara T. (2010). Effects of acute hypoxia at moderate altitude on stroke volume and cardiac output during exercise. Int. Heart J..

[3] Richalet J.P., Hermand E., Lhuissier F.J. (2024). Cardiovascular physiology and pathophysiology at high altitude. Nat. Rev. Cardiol..

[4] Holloway C.J., Montgomery H.E., Murray A.J., Cochlin L.E., Codreanu I., Hopwood N., Johnson A.W., Rider O.J., Levett D.Z., Tyler D.J. (2011). Cardiac response to hypobaric hypoxia: Persistent changes in cardiac mass, function, and energy metabolism after a trek to Mt. Everest Base Camp. FASEB J..

[5] Pena E., Brito J., El Alam S., Siques P. (2020). Oxidative Stress, Kinase Activity and Inflammatory Implications in Right Ventricular Hypertrophy and Heart Failure under Hypobaric Hypoxia. Int. J. Mol. Sci..

[6] Holdsworth D.A., Frise M.C., Bakker-Dyos J., Boos C., Dorrington K.L., Woods D., Mellor A., Robbins P.A. (2020). Iron bioavailability and cardiopulmonary function during ascent to very high altitude. Eur. Respir. J..

[7] Huez S., Faoro V., Guenard H., Martinot J.B., Naeije R. (2009). Echocardiographic and tissue Doppler imaging of cardiac adaptation to high altitude in native highlanders versus acclimatized lowlanders. Am. J. Cardiol..

[8] El Alam S., Pena E., Aguilera D., Siques P., Brito J. (2022). Inflammation in Pulmonary Hypertension and Edema Induced by Hypobaric Hypoxia Exposure. Int. J. Mol. Sci..

[9] Ostadal B., Kolar F. (2007). Cardiac adaptation to chronic high-altitude hypoxia: Beneficial and adverse effects. Respir. Physiol. Neurobiol..

[10] Brito J., Siques P., Leon-Velarde F., De La Cruz J.J., Lopez V., Herruzo R. (2007). Chronic intermittent hypoxia at high altitude exposure for over 12 years: Assessment of hematological, cardiovascular, and renal effects. High. Alt. Med. Biol..

[11] Zhang Q., Wang J., Zhu L., Jiang S.J., Liu J., Wang L.X., Qin X.H. (2021). Ligustrazine Attenuates Hyperhomocysteinemia-induced Alzheimer-like Pathologies in Rats. Curr. Med. Sci..

[12] Feng F., Xu D.Q., Yue S.J., Chen Y.Y., Tang Y.P. (2024). Neuroprotection by tetramethylpyrazine and its synthesized analogues for central nervous system diseases: A review. Mol. Biol. Rep..

[13] Zuo Z., Zuo P.F., Sheng Z.L., Wang X., Ding J.D., Ma G.S. (2019). Tetramethylprazine attenuates myocardial ischemia/reperfusion injury through modulation of autophagy. Life Sci..

[14] Wang G.F., Shi C.G., Sun M.Z., Wang L., Wu S.X., Wang H.F., Xu Z.Q., Chen D.M. (2013). Tetramethylpyrazine attenuates atherosclerosis development and protects endothelial cells from ox-LDL. Cardiovasc. Drugs Ther..

[15] Li Y.J., Liu R.P., Ding M.N., Zheng Q., Wu J.Z., Xue X.Y., Gu Y.Q., Ma B.N., Cai Y.J., Li S. (2022). Tetramethylpyrazine prevents liver fibrotic injury in mice by targeting hepatocyte-derived and mitochondrial DNA-enriched extracellular vesicles. Acta Pharmacol. Sin..

[16] Lin K.H., Kuo W.W., Jiang A.Z., Pai P., Lin J.Y., Chen W.K., Day C.H., Shen C.Y., Padma V.V., Huang C.Y. (2015). Tetramethylpyrazine Ameliorated Hypoxia-Induced Myocardial Cell Apoptosis via HIF-1alpha/JNK/p38 and IGFBP3/BNIP3 Inhibition to Upregulate PI3K/Akt Survival Signaling. Cell Physiol. Biochem..

[17] Yu B., Zhong F.M., Yao Y., Deng S.Q., Xu H.Q., Lu J.F., Ruan M., Shen X.C. (2019). Synergistic protection of tetramethylpyrazine phosphate and borneol on brain microvascular endothelium cells injured by hypoxia. Am. J. Transl. Res..

[18] Hoelz A., Nairn A.C., Kuriyan J. (2003). Crystal structure of a tetradecameric assembly of the association domain of Ca^2+^/calmodulin-dependent kinase II. Mol. Cell.

[19] Swaminathan P.D., Purohit A., Hund T.J., Anderson M.E. (2012). Calmodulin-dependent protein kinase II: Linking heart failure and arrhythmias. Circ. Res..

[20] Ling H., Zhang T., Pereira L., Means C.K., Cheng H., Gu Y., Dalton N.D., Peterson K.L., Chen J., Bers D. (2009). Requirement for Ca^2+^/calmodulin-dependent kinase II in the transition from pressure overload-induced cardiac hypertrophy to heart failure in mice. J. Clin. Investig..

[21] Zhang T., Maier L.S., Dalton N.D., Miyamoto S., Ross J., Bers D.M., Brown J.H. (2003). The deltaC isoform of CaMKII is activated in cardiac hypertrophy and induces dilated cardiomyopathy and heart failure. Circ. Res..

[22] Zhang T., Zhang Y., Cui M., Jin L., Wang Y., Lv F., Liu Y., Zheng W., Shang H., Zhang J. (2016). CaMKII is a RIP3 substrate mediating ischemia- and oxidative stress-induced myocardial necroptosis. Nat. Med..

[23] Joiner M.L., Koval O.M., Li J., He B.J., Allamargot C., Gao Z., Luczak E.D., Hall D.D., Fink B.D., Chen B. (2012). CaMKII determines mitochondrial stress responses in heart. Nature.

[24] He B.J., Joiner M.L., Singh M.V., Luczak E.D., Swaminathan P.D., Koval O.M., Kutschke W., Allamargot C., Yang J., Guan X. (2011). Oxidation of CaMKII determines the cardiotoxic effects of aldosterone. Nat. Med..

[25] Zhang R., Khoo M.S., Wu Y., Yang Y., Grueter C.E., Ni G., Price E.E., Thiel W., Guatimosim S., Song L.S. (2005). Calmodulin kinase II inhibition protects against structural heart disease. Nat. Med..

[26] van Oort R.J., McCauley M.D., Dixit S.S., Pereira L., Yang Y., Respress J.L., Wang Q., De Almeida A.C., Skapura D.G., Anderson M.E. (2010). Ryanodine receptor phosphorylation by calcium/calmodulin-dependent protein kinase II promotes life-threatening ventricular arrhythmias in mice with heart failure. Circulation.

[27] Mesubi O.O., Anderson M.E. (2016). Atrial remodelling in atrial fibrillation: CaMKII as a nodal proarrhythmic signal. Cardiovasc. Res..

[28] Sossalla S., Fluschnik N., Schotola H., Ort K.R., Neef S., Schulte T., Wittkopper K., Renner A., Schmitto J.D., Gummert J. (2010). Inhibition of elevated Ca^2+^/calmodulin-dependent protein kinase II improves contractility in human failing myocardium. Circ. Res..

[29] Khoo M.S., Li J., Singh M.V., Yang Y., Kannankeril P., Wu Y., Grueter C.E., Guan X., Oddis C.V., Zhang R. (2006). Death, cardiac dysfunction, and arrhythmias are increased by calmodulin kinase II in calcineurin cardiomyopathy. Circulation.

[30] Backs J., Backs T., Neef S., Kreusser M.M., Lehmann L.H., Patrick D.M., Grueter C.E., Qi X., Richardson J.A., Hill J.A. (2009). The delta isoform of CaM kinase II is required for pathological cardiac hypertrophy and remodeling after pressure overload. Proc. Natl. Acad. Sci. USA.

[31] Reyes Gaido O.E., Nkashama L.J., Schole K.L., Wang Q., Umapathi P., Mesubi O.O., Konstantinidis K., Luczak E.D., Anderson M.E. (2023). CaMKII as a Therapeutic Target in Cardiovascular Disease. Annu. Rev. Pharmacol. Toxicol..

[32] Feng N., Anderson M.E. (2017). CaMKII is a nodal signal for multiple programmed cell death pathways in heart. J. Mol. Cell Cardiol..

[33] Luczak E.D., Wu Y., Granger J.M., Joiner M.A., Wilson N.R., Gupta A., Umapathi P., Murphy K.R., Reyes Gaido O.E., Sabet A. (2020). Mitochondrial CaMKII causes adverse metabolic reprogramming and dilated cardiomyopathy. Nat. Commun..

[34] Tenma T., Mitsuyama H., Watanabe M., Kakutani N., Otsuka Y., Mizukami K., Kamada R., Takahashi M., Takada S., Sabe H. (2018). Small-conductance Ca(2+)-activated K(+) channel activation deteriorates hypoxic ventricular arrhythmias via CaMKII in cardiac hypertrophy. Am. J. Physiol. Heart Circ. Physiol..

[35] Fu C., Hao J., Zeng M., Song Y., Jiang W., Zhang P., Luo A., Cao Z., Belardinelli L., Ma J. (2017). Modulation of late sodium current by Ca(2+) -calmodulin-dependent protein kinase II, protein kinase C and Ca(2+) during hypoxia in rabbit ventricular myocytes. Exp. Physiol..

[36] Pelouch V., Kolar F., Ost’adal B., Milerova M., Cihak R., Widimsky J. (1997). Regression of chronic hypoxia-induced pulmonary hypertension, right ventricular hypertrophy, and fibrosis: Effect of enalapril. Cardiovasc. Drugs Ther..

[37] Chan C.S., Lin F.J., Chen Y.C., Lin Y.K., Higa S., Chen S.A., Chen Y.J. (2023). Glucagon-like Peptide-1 Receptor Activation Reduces Pulmonary Vein Arrhythmogenesis and Regulates Calcium Homeostasis. Int. J. Mol. Sci..

[38] Yang Q., Huang D.D., Li D.G., Chen B., Zhang L.M., Yuan C.L., Huang H.H. (2019). Tetramethylpyrazine exerts a protective effect against injury from acute myocardial ischemia by regulating the PI3K/Akt/GSK-3beta signaling pathway. Cell Mol. Biol. Lett..

[39] Feng J., Wu G., Tang S. (1999). The effects of tetramethylpyrazine on the incidence of arrhythmias and the release of PGI2 and TXA2 in the ischemic rat heart. Planta Med..

[40] Lu D., Shao H.T., Ge W.P., Liu N., Zhang X., Ma C.M., Qin C., Zhang L.F. (2012). Ginsenoside-Rb1 and tetramethylpyrazine phosphate act synergistically to prevent dilated cardiomyopathy in cTnTR141W transgenic mice. J. Cardiovasc. Pharmacol..

[41] Zhu X., Wang K., Zhang K., Tan X., Wu Z., Sun S., Zhou F., Zhu L. (2015). Tetramethylpyrazine Protects Retinal Capillary Endothelial Cells (TR-iBRB2) against IL-1beta-Induced Nitrative/Oxidative Stress. Int. J. Mol. Sci..

[42] Lin J., Wang Q., Zhou S., Xu S., Yao K. (2022). Tetramethylpyrazine: A review on its mechanisms and functions. Biomed. Pharmacother..

[43] Li G., Sng K.S., Shu B., Wang Y.J., Yao M., Cui X.J. (2023). Effects of tetramethylpyrazine treatment in a rat model of spinal cord injury: A systematic review and meta-analysis. Eur. J. Pharmacol..

[44] Mazzeo R.S., Reeves J.T. (2003). Adrenergic contribution during acclimatization to high altitude: Perspectives from Pikes Peak. Exerc. Sport. Sci. Rev..

[45] Beghi S., Furmanik M., Jaminon A., Veltrop R., Rapp N., Wichapong K., Bidar E., Buschini A., Schurgers L.J. (2022). Calcium Signalling in Heart and Vessels: Role of Calmodulin and Downstream Calmodulin-Dependent Protein Kinases. Int. J. Mol. Sci..

[46] Pang P.K., Shan J.J., Chiu K.W. (1996). Tetramethylpyrazine, a calcium antagonist. Planta Med..

[47] Tsai C.C., Lai T.Y., Huang W.C., Yang T., Liu I.M., Wong K.L., Chan P., Cheng J.T. (2003). Tetramethylpyrazine as potassium channel opener to lower calcium influx into cultured aortic smooth muscle cells. Planta Med..

[48] Ren Z., Ma J., Zhang P., Luo A., Zhang S., Kong L., Qian C. (2012). The effect of ligustrazine on L-type calcium current, calcium transient and contractility in rabbit ventricular myocytes. J. Ethnopharmacol..

[49] Picca A., Mankowski R.T., Burman J.L., Donisi L., Kim J.S., Marzetti E., Leeuwenburgh C. (2018). Mitochondrial quality control mechanisms as molecular targets in cardiac ageing. Nat. Rev. Cardiol..

[50] Nouette-Gaulain K., Malgat M., Rocher C., Savineau J.P., Marthan R., Mazat J.P., Sztark F. (2005). Time course of differential mitochondrial energy metabolism adaptation to chronic hypoxia in right and left ventricles. Cardiovasc. Res..

[51] Mallet R.T., Burtscher J., Pialoux V., Pasha Q., Ahmad Y., Millet G.P., Burtscher M. (2023). Molecular Mechanisms of High-Altitude Acclimatization. Int. J. Mol. Sci..

[52] Luczak E.D., Anderson M.E. (2014). CaMKII oxidative activation and the pathogenesis of cardiac disease. J. Mol. Cell Cardiol..

[53] Omura J., Habbout K., Shimauchi T., Wu W.H., Breuils-Bonnet S., Tremblay E., Martineau S., Nadeau V., Gagnon K., Mazoyer F. (2020). Identification of Long Noncoding RNA H19 as a New Biomarker and Therapeutic Target in Right Ventricular Failure in Pulmonary Arterial Hypertension. Circulation.

