# Supplementary figures and images for: Tetramethylpyrazine Protects Against Chronic Hypobaric Hypoxia-Induced Cardiac Dysfunction by Inhibiting CaMKII Activation in a Mouse Model Study
Source: Int J Mol Sci. 2024 Dec 24;26(1):54. doi: 10.3390/ijms26010054 (PMC11720575; doi:10.3390/ijms26010054)

Figure 5A

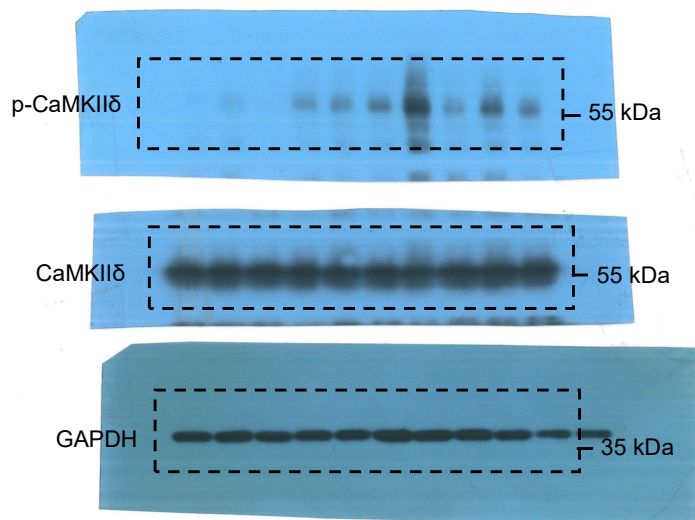

Figure 5B

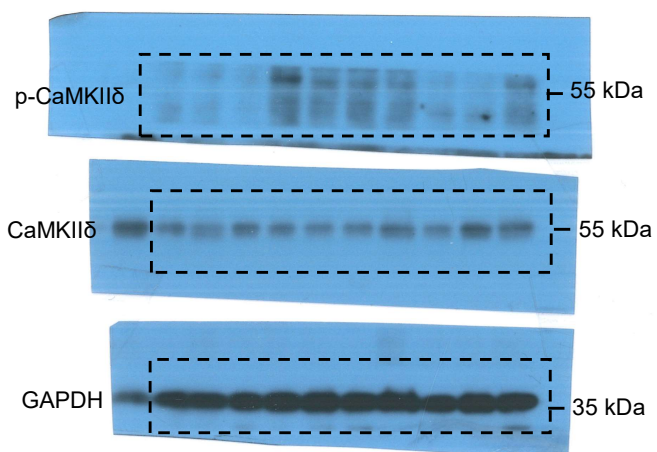

Supplement: Supplementary file 1 [file ijms-26-00054-s001.zip › ijms-3361999-supplementary.pdf]
